# Speciality interests and career calling to medicine among first-year medical students

**DOI:** 10.1007/s40037-012-0037-9

**Published:** 2013-01-09

**Authors:** Nicole J. Borges, R. Stephen Manuel, Ryan D. Duffy

**Affiliations:** 1 Wright State University Boonshoft School of Medicine, Academic Affairs, 290P White Hall, Dayton, OH 45401-0927 USA; 2 University of Cincinnati College of Medicine, Cincinnati, OH USA; 3 University of Florida, Gainesville, FL USA

**Keywords:** Speciality, Calling, Medical students, Career

## Abstract

The construct of calling has recently been applied to the vocation of medicine. We explored whether medical students endorse the presence of a calling or a search for a calling and how calling related to initial speciality interest. 574 first-year medical students (84 % response rate) were administered the Brief Calling Survey and indicated their speciality interest. For presence of a calling, the median response was *mostly* true for: ‘I have a calling to a particular kind of work’ and *moderately* true for: ‘I have a good understanding of my calling as it applies to my career’. For search for a calling, median response was *mildly* true: ‘I am trying to figure out my calling in my career’ and ‘I am searching for my calling as it applies to my career’. Mann–Whitney *U* (*p* < 0.05) results indicate that students interested in primary care (*n* = 185) versus non-primary care (*n* = 389) are more likely to endorse the presence of a calling. Students were more likely to endorse the presence of a calling rather than a search for a calling, with those interested in primary care expressing stronger presence of a calling to medicine.

## Introduction

‘Calling’ to a vocation and its connections to career development and wellbeing is a growing area of scholarly inquiry. Reviewing dozens of prior definitions, Dik and Duffy [1] defined a calling as a career that (a) involves an external summons, (b) provides a sense of meaning or purpose, and (c) is used to help others in some capacity. With samples of working adults and college students, viewing one’s career in such a fashion has been linked to positive work and wellbeing outcomes, including increased career maturity, academic satisfaction, job satisfaction, career commitment, life meaning, and life satisfaction [2–5].

Given that research on calling is emerging, no studies to date have compared individuals in one profession with another regarding endorsement of a calling. Although calling is a term frequently used colloquially within the field of medicine, only two studies to date have examined the construct with physicians or medical students. Duffy et al. [6] surveyed a group of medical students prior to the start of their first and third years, and found that over time feeling called was correlated with increased life meaning and career maturity. Bott et al. [7] interviewed physicians in academic medicine who felt their career was a calling and found that these individuals were highly satisfied with their work and life, and many felt called to multiple components of their job, including research, teaching, and practice.

The paucity of research on calling in the field of medicine leaves a great deal of questions to be investigated. The purpose of the current study is twofold. First, we sought to answer the question of whether medical students endorse the *presence of a calling* or a *search for a calling* when they first begin medical school and, second, we explored whether medical students who endorse an interest in primary care versus non-primary care specialities when they first begin medical school differ regarding having the presence of, or search for, a career calling.

## Methods

With institutional review board approval, 574 first-year medical students (84 % response rate) at one United States medical school were administered the Brief Calling Survey (BCS) [8] when they started medical school in 2008 (*n* = 111), 2009 (*n* = 148), 2011 (*n* = 172), and 2012 (*n* = 143). (The BCS was not administered in 2010 because of scheduling difficulties with survey administration). The BCS has been used in several studies previously with findings to support its reliability and correlations with career maturity. This 4-item survey uses a Likert-type response scale: *Not at all true of me* (1); *Mildly true of me* (2); *Moderately true of me* (3); *Mostly true of me* (4); *Totally true of me* (5). The four items are as follows: ‘I have a calling to a particular kind of work’, ‘I have a good understanding of my calling as it applies to my career’, ‘I am trying to figure out my calling in my career’, and ‘I am searching for my calling as it applies to my career’. Scholars have pointed out that perceiving a calling and searching for a calling are not mutually exclusive, as people could have a calling in one area but be searching for a different calling, or could be searching for more than one calling [1]. This distinction is evident in data for the current study, as the two presence items only correlated between 0.05 and 0.16 with the two search items, indicating these are distinct constructs. These students also indicated their speciality interest. For purposes of data analysis, specialities were coded as primary versus non-primary care.

## Results

Findings suggest that first-year medical students were more likely to endorse the presence of a calling compared with the search for a calling (Table 1). Regarding the presence of a calling, Mann–Whitney *U* results indicate that first-year medical students interested in primary care (*n* = 185) versus non-primary care (*n* = 389) were more likely to endorse the presence of a calling item (*p* < 0.05): ‘I have a calling to a particular kind of work’ (*U* = 30907, *z* = −2.69, *p* = < 0.01, *r* = 0.11). Effect size was small. No significant differences between the two groups existed for ‘I have a good understanding of my calling as it applies to my career’ (*U* = 32633, *z* = −1.82, *p* = 0.07). For search for a calling, no significant differences existed between groups regarding ‘I am trying to figure out my calling in my career’ (*U* = 33842, *z* = −0.99, *p* = 0.32) and ‘I am searching for my calling as it applies to my career’ (*U* = 34582, *z* = −0.73, *p* = 0.47).

**Table 1:** Frequency and percentages for Brief Calling Survey

	Presence of a calling		Search for a calling	
	I have a calling to a particular kind of work	I have a good understanding of my calling as it applies to my career	I am trying to figure out my calling in my career	I am searching for my calling as it applies to my career
Median	4	3	2	2
Interquartile range	2–4	2–4	2–4	2–3
Not at all true of me (1)	53 (9.2 %)	58 (10.1 %)	111 (19.3 %)	126 (22 %)
Mildly true of me (2)	90 (15.7 %)	108 (18.8 %)	191 (33.3 %)	171 (29.8 %)
Moderately true of me (3)	116 (20.2 %)	167 (29.1 %)	124 (21.6 %)	135 (23.5 %)
Mostly true of me (4)	212 (36.9 %)	186 (32.4 %)	104 (18.1 %)	98 (17.1 %)
Totally true of me (5)	103 (17.9 %)	55 (9.6 %)	43 (7.5 %)	44 (7.7 %)

## Conclusion

The results from this study shed important light on the construct of calling within first-year medical students. Generally, first-year medical students endorsed having a calling, and were more likely to endorse having a calling than searching for one. These results suggest that calling is a salient construct with regard to how first-year medical students view their career. Additionally, at this point in their career development, students may feel strongly that medicine is the career they are called to, and thus are less likely to be searching for a calling. Students embarking on a career in medicine who are interested in primary care also expressed a stronger presence of a calling compared with students interested in non-primary care specialities. Given that a core component of calling is prosocial career motivation, or more simply a motivation to help others through one’s work, primary care specialities may offer more ample opportunities to directly help others. As such, students interested in primary care specialities may be more likely to think of their career as a calling.
